# Hemizygous contiguous gene deletion within Xq28 that includes BCAP31, ABCD1, SRPK3 and SSR4: case report and literature review

**DOI:** 10.1016/j.gmg.2025.100066

**Published:** 2025-06-21

**Authors:** Joan Chern-Hui Lien, Vinay D. Chandrasekaran, Tarachandra M. Narumanchi, Melissa S. Napoli, Jo Elle G. Peterson, Charles A. Williams

**Affiliations:** a Section of Neonatal-Perinatal Medicine, University of Oklahoma College of Medicine, Oklahoma City, OK, USA; b Genetics Specialty Clinic, Children’s Hospital, Oklahoma University Health Science Center, Oklahoma City, OK, USA; c Department of Pathology, University of Oklahoma College of Medicine, Oklahoma City, OK, USA; d Division of Genetics and Metabolism, Department of Pediatrics, University of Florida College of Medicine, Gainesville, FL, USA

**Keywords:** Contiguous gene deletion, Xq28, X chromosome, Microarray

## Abstract

We report on a rare 110 kilobase contiguous gene deletion within chromosome region Xq28, encompassing 7 annotated Online Mendelian Inheritance in Man (OMIM) genes and extending from BCAP31 to the telomeric-located PDZD4. We review 13 other reported contiguous deletions in this region and analyze their clinical phenotypes. The novel findings in our case were orofacial clefting and a vascular ring. The major clinical anomalies in our case appear to be due to the combined effects of BCAP31, SRPK3 and SSR4 deletions. This combination produces a severe neonatal disorder whose features further refine our knowledge about deletions within Xq28. Additionally, the observation of multiple nonrecurring breakpoints among the published cases suggests that the deletions occur by random chromosome breakage.

## Graphical abstract

**Figure:**
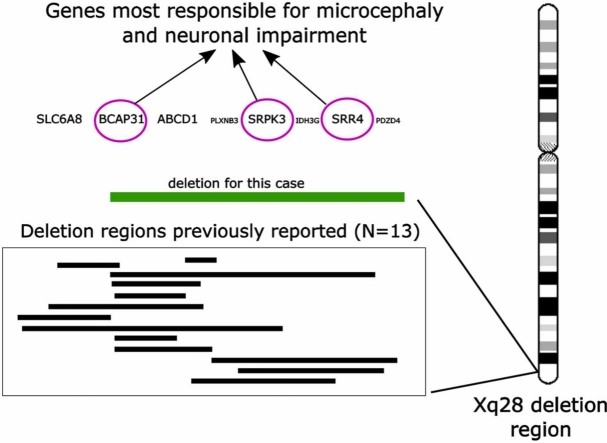

## Introduction

Unlike most relatively small genomic deletions on autosomes, deletions on X chromosomes typically cause deleterious effects, often due to contiguous gene disruption. Within the deletion boundaries of our case, previous reports have mainly discussed the biochemical and clinical effects related to disruption or deletion of ABCD1 and BCAP31.

ABCD1 encodes an ATP-binding cassette (ABC) protein transporter that functions in peroxisomal metabolism of very long chain fatty acids [1]. ABCD1 mutations are well known to cause X-linked adrenal leukodystrophy. Newborns are asymptomatic but express the fatty acid biochemical phenotype, identified upon newborn blood spot screening.

BCAP31 (B-cell receptor-associated protein 31, OMIM #300398) is a chaperone protein highly expressed in the brain and involved in crosstalk between the endoplasmic reticulum and the Golgi apparatus. Approximately 30 cases have been reported, mainly involving intragenic loss of function or missense mutations, but also including larger deletions encompassing contiguous genes, most involving ABCD1 [2], [3], [4], [5], [6]. Disruption of BCAP31 alone causes congenital deafness, dystonia, and cerebral hypomyelination (DDCH syndrome) [4]. Hepatic dysfunction and microcephaly are also described. Contiguous gene deletions that include ABCD1 have been termed CADDS (Contiguous ABCD1 DXS1375 E Deletion Syndrome) but the major phenotype of CADDS is due to BCAP31 disruption [6], [7], [8].

We report a case involving a larger deletion, including BCAP31 and ABCD1, and extending toward the telomere to include SRPK3 and SSR4, genes that have only recently been identified as causing a clinical phenotype.

## Case presentation

A severely growth-restricted male infant was delivered at 35 6/7 weeks’ gestational age to a 17-year-old G1 mother via emergent Cesarean section after persistent non-reassuring fetal heart tracings. Prenatal findings included oligohydramnios, severe fetal growth restriction, right-sided aortic arch with vascular ring, midline arachnoid cyst, and cleft lip and palate. Amniocentesis revealed microdeletion in region Xq28.

**Fig. 1:**
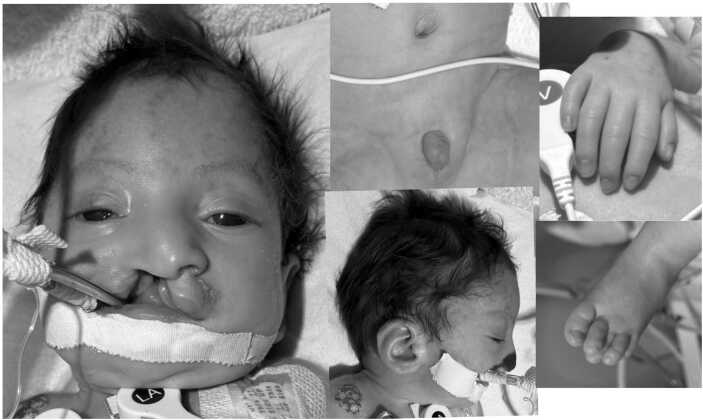
Composite photo of patient, showing microcephaly, clef lip and palate, hypertelorism, large ears, micropenis, undescended testes, relatively thin fingers and overlapping toes.

Birth weight was 1260 g with length and head circumference of 39.5 cm and 27.4 cm, respectively (all measurements <1 % for gestational age). He was admitted to the NICU for respiratory support and further evaluation of sequelae secondary to his genetic deletion. Physical exam was notable for a growth restricted, microcephalic male infant with a large right-sided cleft lip and palate, micrognathia, telecanthus, eyelid ptosis, large low-set ears, micropenis, empty scrotal sac, and generalized hypotonia (Fig. 1). The fingers were relatively long and thin, with long and narrow feet with overlapping toes.

Echocardiography on day two of life showed a small patent foramen ovale, small anterior muscular ventricular septal defect, right aortic arch with aberrant left subclavian artery consistent with vascular ring, and normal biventricular size and function.

Early cranial ultrasound revealed small bilateral grade 1 germinal matrix hemorrhages, absent corpus callosum, and mild lateral ventriculomegaly. There was also evidence of gliosis in the subcortical white matter and right cerebellar hemisphere. Repeat imaging noted periventricular leukomalacia, subependymal heterotopia, bilateral lenticulostriate vasculopathy, and absent corpus callosum.

His hospital course was complicated by respiratory failure, feeding intolerance, severe growth retardation (Z scores consistently < 5), persistent neutrophilia (WBC 30–65 ×103/μL, ANC 11–40 ×103/μL) without obvious source of infection, autonomic dysfunction with temperature instability and heart rate fluctuations. There was neuro-irritability with difficulty weaning off sedation medications. Other problems included central line associated deep vein thromboses, growth hormone deficiency (IGFBP3 0.73 mg/L), mild liver dysfunction (peak values: AST 186 U/L, ALT 200 U/L, direct bilirubin 3.8 mg/dL, GGT 891 U/L), osteopenia (peak alkaline phosphatase 796 U/L), asymptomatic vascular ring, and bilateral small kidneys with grade 1 dilatation.

At 3 months of age, he developed fever, abdominal distension, bloody stools and an x-ray showed pneumatosis intestinalis. He expired shortly after despite resuscitation attempts. Blood cultures later grew *Klebsiella oxytoca*, *Escherichia coli*, and *Enterococcus faecalis*. Autopsy confirmed necrotizing enterocolitis as cause of death. Gross examination of the brain showed it to be well-formed but notably smaller than expected for the patient's age, with a fixed weight of 330 g compared to the expected 567.0 ± 81.0 g. The primary gross finding was significant hypoplasia of the corpus callosum, which measured less than 1 mm in thickness in some regions. Additionally, bilateral ventral Probst [9] bundles were observed, extending the entire length of the corpus callosum. These bundles were located parasagittal in the anterior region and progressively shifted laterally towards the posterior aspect. No gross malformations, heterotopias, or germinal matrix hemorrhages were detected. Microscopic examination revealed nonspecific changes, such as microglial activation and generalized edema, but no other significant pathological findings were noted.

## Genetic testing

Newborn blood spot screening showed abnormal elevation of very long chain fatty acids with C26:0 at 0.92 µmol/L (normal range < 0.58), with repeat screen revealing similar results (0.93).

Postnatal blood testing showed a 46, XY karyotype while chromosomal microarray study revealed a novel de novo 130 Kilobase deletion (arr [GRCh37]; 152976269_153106700) within Xq28 involving 7 OMIM genes: BCAP31, ABCD1, PLXNB3, SRPK3, ICH3G, SSR4 and PDZD4. The mother’s chromosome microarray was normal. A trio whole exome sequencing study also identified a maternally inherited, in Titin (TTN: p. R15019 *). This mutation was classified as likely pathogenic.

The patient’s family history showed no occurrences of cardiomyopathy or other genetic disorders or congenital anomalies including cleft palate, congenital urologic abnormalities, and vascular malformations. The mother was apparently healthy with no history of heart disease.

## Discussion

**Fig. 2:**
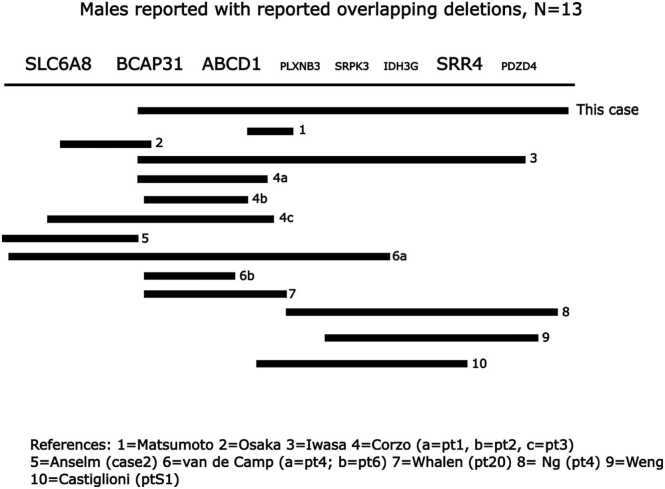
Gene deletion map of current case patient compared to male patients presented in literature with overlapping continuous gene deletions.

We present a case involving the deletions of BCAP31, ABCD1, SRPK3, and SSR4. Another case report by Iwasa et. al in 2013 [7] also reported deletion of these 4 genes (Fig. 2), but at the time of that publication, no disease phenotypes had been linked to SRPK3 or SSR4. We now have greater insight into the clinical effects associated with these additional two genes.

SRPK3 (serine/arginine [SR] specific protein kinase 3) is one of a family of kinases that phosphorylate SR repeat-containing proteins. These proteins can then enter the nucleus and act as splicing factors [10]. Recently, Roychaudhury et al., identified missense variants and a putative truncating variant in SRPK3 in 9 X-linked intellectual deficiency (XLID) patients from 5 unrelated families [11]. All showed global developmental delay but some also exhibited ataxia; none had facial dysmorphism. MRI findings were variable and included severe white matter and cortical anomalies, colpocephy and under development of the corpus callosum and cerebellar hypoplasia. Several families had normal brain MRI. Slender fingers were reported in two families. The authors felt that hypomorphic or truncating alleles accounted for the phenotype. However, we found no reports of solitary SRPK3 gene deletions but the above studies suggest that whole gene deletion would lead to an abnormal neurodevelopmental phenotype.

Signal sequence receptor protein 4 (SSR4) encodes the delta subunit of the translocation-associated protein (TRAP), a hetero-tetrameric complex. This complex localizes on the membrane of the endoplasmic reticulum, is involved in secretion of proteins and plays a role in posttranslational N-glycosylation. Disruption of SSR4 is now known to cause congenital disorder of glycosylation type 1 y (CDG1Y; OMIM #300934) an X-linked disorder characterized by developmental delay, muscular hypotonia, microcephaly, and distinctive facial features. Less commonly, patients can present with epilepsy, skeletal abnormalities, and connective tissue disease [12], [13], [14].

We summarized the clinical features observed in all the reported contiguous gene cases (Table 1) [2], [3], [4], [6], [7], [14], [15], [16], [17], [18]. Fig. 2 illustrates the deletion regions of these cases, and the occurrence of multiple nonrecurring breakpoints suggests that the deletions occur by random chromosome breakage. After our review, it appeared that the clinical features associated with SRPK3 disruption, including brain imaging findings, and the relatively large ears and thin fingers, seen in both SRPK3 and SSR4 cases, probably contribute to our patient’s phenotype, in addition to the effects of ABCD1 and BCAP31 deletion.

**Table 1:** Clinical findings in overlapping contiguous gene deletion cases found in literature and in the reported patient.

**Features**	**Matsumoto 2005**	**Osaka 2012**	**Iwasa 2013**	**Corzo 2002** **“Pt 1″**	**Corzo 2002** **“Pt 2″**	**Corzo 2002** **“Pt 3″**	**Anselm 2006** **“Case 2″**	**Van de Kamp 2014** **“Pt 4″**	**Van de Kamp 2014** **“Pt 6″**	**Whalen 2021** **“Pt 20″**	**Weng 2023**	**Castiglioni 2020** **“S1″**	**Ng** **2015** **“Pt P4″**	**Current Patient**
GDD	-	+	+	U	U	U	+	U	+	+	+	+	+	U
IUGR or FTT	-	+	+	+	+	+	+	U	+	+	+	+	U	+
Abnormal Tone and/or Dystonia	-	+	+	+	+	+	+	+	+	+	+	+	+	+
Seizures	+	+	U	-	+	+	+	U	-	-	-	-	U	-
Liver Disease	U	+	+	+	+	+	U	+	+	+	U	U	U	+
Blindness or Retinal Abnormalities	U	U	U	+	-	-	+	U	-	+	U	-		-
SNHL or Deafness	U	+	+	+	+	-	+	+	U	-	+	-	U	U
Early Death (Age)	U	U	+ (8mo)	+ (11mo)	+ (4mo)	+ (4mo)	U	+ (5mo)	+ (8mo)	+ (16mo)	U	U	U	+ (3mo)
Microcephaly	U	U	+	U	U	U	+	U	+	+	-	+	+	+
Brain Imaging	MRI/FLAIR – High-intensity areas in temporal and frontal cerebrum	MRI/T2 – Increased signals in globus pallidi, myelination delay, decrease in cerebral white matter volume, thin corpus callosum	Autopsy – Small brain w/ hypomyelination, white matter heterotopia and leptomeningeal glioneuronal heterotopia	U	U	Autopsy – White matter abnormalities, myelination delay	MRI – High-intensity areas in globus pallidi, delayed white matter myelination delay, thin corpus callosum	MRI – Mild focal dilatation left Sylvian fissure, immature myelination	MRI – No abnormalities	MRI – High-intensity areas in thalami	U	MRI – No abnormalities	U	HUS – Agenesis of corpus callosum
Dysmorphic Features	U	-	+	-	-	-	+	U	+	U	+	+	+	+
Description of Dysmorphia			Flat orbital edge, hypoplastic nose, micrognathia, inguinal hernia, micropenis, cryptorchidism, club feet				Prominent nasal bridge, high-arched palate w/ submucus cleft, 5th finger clinodactyly		Not described			Broad nasal bridge, long philtrum, micrognathia, 5th finger clinodactyly, cryptorchidism	Not described	Cleft lip and palate, telecanthus, low-set ears, microphallus, cryptorchidism

Abbreviations: GDD = global developmental delay, IUGR = intrauterine growth restriction, FTT = failure to thrive, SNHL = sensorineural hearing loss, mo = months old, MRI = magnetic resonance imaging, HUS = head ultrasound, (+) = Feature present, (-) = Feature absent, (U) = Feature not reported or unknown

This infant showed several clinical problems that could be related to SSR4 deletion. The thrombosis events could be related to a coagulopathy that has been well-described in carbohydrate glycosylation disorders [12], [19], [20] as has growth hormone deficiency [19].

The transient bilirubin elevations and ongoing elevation of liver enzymes (e.g. ALT) are likely related to the effect of the BPCAP31 deletion although other factors could be contributory [6]. The problems of persistent neutrophilia and osteopenia remain unexplained and may be unrelated to the effects of gene deletions in the region.

No previously reported case had orofacial clefting. No apparent cause for the cleft in our patient was identified with either array chromosome or whole exome testing. The prenatal exposure history only involved daily Valtrex, for HSV-1 suppression. Valtrex appears to have no increased risk for facial clefts or other birth defects[21]. Mother tested negative for other infectious diseases, including human immunodeficiency virus, syphilis, Toxoplasmosis, and cytomegalovirus. Previous linkage studies have identified polymorphisms on the X chromosome that confer risk for orofacial clefts but none of those polymorphisms mapped within our deletion region[22]. To what extent the combined effects of the deleted genes, or the regulatory effects of deleted noncoding areas in the region, contributed to causing the facial cleft is unclear.

The other genes deleted in our patient (PLXNB3, ICH3G, and PDZD4) appear to be of lesser significance. PLXNB3 encodes a large transmembrane protein and is part of a family of receptors for transmembrane, secreted, and glycosylphosphatidylinositol (GPI)-anchored semaphorins [23]. The intracellular signals transduced by plexins are largely obscure. Haplotype analysis of PLXNB3 polymorphisms in 303 volunteers and 42 males found an assocation with increased verbal ability and brain volume [24]. Recently, an association of congenital heart disease and neurodevelopmental problems was linked to PLXNB3 missense mutations [25]. We found no reports of solitary PLXNB3 deletions. IDH3G encodes a subunit of a heterotetrameric enzyme that is located in mitochondria and presumed to play a major role in the oxidative decarboxylation of isocitrate [26]. We could find no clinical case reports of IDH3G mutations or deletions. PDZD4 encodes a 769-amino acid protein containing a bipartite nuclear localization signal and a PDZ domain; it is highly expressed in the brain but its role in brain function is unclear [27]. In a screen for skewed X-chromosome inactivation in unsolved neurodevelopmental disorders, a boy was found to have a missense mutation predicted to impair protein function. He was followed for kyphoscoliosis with pectus excavatum, hyperelastic skin and joints, persistent hand tremors and facial dysmorphisms and polymicrogyria [28]. Also, in a 285 person cohort with autism spectrum disorder (ASD) or schizophrenia, a male with ASD was found to have a significant PDZD4 missense mutation [29], [30]. We found no case of solitary complete PDZD4 deletion.

Whole exome sequencing also identified a secondary variant: a heterozygous truncating mutation in TTN [Titin]. Such heterozygous variants are associated with several adult-onset myopathies and cardiomyopathies [31]. Most commonly, damaging mutations confer an increased risk of dilated cardiomyopathy (DCM) and TTN accounts for 20 %−25 % of familial DCM cases [31]. It is estimated that 0.36 % to 0.5 % of the population carry a pathogenic, DCM-associated mutation [32]. TTN is thus one of the most common genes showing secondary findings in exome sequencing. Outcomes of asymptomatic carriers of TTN mutations are not well understood due to TTN being associated with multiple phenotypes, incomplete penetrance, and variable expressivity. In our case, genetic and surveillance counseling was provided to the mother, the currently asymptomatic carrier.

In summary, we report on a patient having a small contiguous gene deletion within a critical chromosome Xq28 region. The deletion boundaries for our case, and those of the other reported cases, encompass genes critical to normal neurodevelopment. Because ABCD1 maps within this region, neonatal detection of ABCD1 deletion often occurs when newborn screening identifies a presumptive positive for X-linked adrenoleukodystrophy. Because of the rarity of gene deletions in this region, this case report and summary of the other similar cases provide important clinical information for practitioners who encounter similar microdeletions.
